# Triglyceride-Rich Lipoprotein Metabolism: Key Regulators of Their Flux

**DOI:** 10.3390/jcm12134399

**Published:** 2023-06-29

**Authors:** Alejandro Gugliucci

**Affiliations:** Glycation, Oxidation and Disease Laboratory, Department of Research, Touro University California, Vallejo, CA 94592, USA; alejandro.gugliucci@gmail.com

**Keywords:** TRL, ANGPTL, apoCIII, chylomicrons, VLDL, LDL, LPL, atherogenesis, remnants apoB100, apoB48

## Abstract

The residual risk for arteriosclerotic cardiovascular disease after optimal statin treatment may amount to 50% and is the consequence of both immunological and lipid disturbances. Regarding the lipid disturbances, the role of triglyceride-rich lipoproteins (TRLs) and their remnants has come to the forefront in the past decade. Triglycerides (TGs) stand as markers of the remnants of the catabolism of TRLs that tend to contain twice as much cholesterol as compared to LDL. The accumulation of circulating TRLs and their partially lipolyzed derivatives, known as “remnants”, is caused mainly by ineffective triglyceride catabolism. These cholesterol-enriched remnant particles are hypothesized to contribute to atherogenesis. The aim of the present narrative review is to briefly summarize the main pathways of TRL metabolism, bringing to the forefront the newly discovered role of apolipoproteins, the key physiological function of lipoprotein lipase and its main regulators, the importance of the fluxes of these particles in the post-prandial period, their catabolic rates and the role of apo CIII and angiopoietin-like proteins in the partition of TRLs during the fast-fed cycle. Finally, we provide a succinct summary of the new and old therapeutic armamentarium and the outcomes of key current trials with a final outlook on the different methodological approaches to measuring TRL remnants, still in search of the gold standard.

## 1. Introduction

The cornerstone for lowering the risk of atherosclerotic cardiovascular disease (ASCVD), for the past three decades, has been statin medication [1,2,3,4,5]. Numerous investigations conducted during this time have conclusively shown a link between low-density lipoprotein cholesterol (LDL-C) and ASCVD [1,3,6,7]. As a result, medications that reduce total lifetime exposure to LDL-C have been effective in reducing the risk of ASCVD. However, despite the effectiveness and affordability of statin medication, ASCVD continues to be the world’s leading cause of death. It is significant that many people using statin therapy still have a residual risk of developing ASCVD and cannot meet their target LDL-C targets [4,8,9,10,11,12,13,14,15,16,17]. This residual risk, which may amount to 50% after optimal statin treatment, is the consequence of both immunological and lipid disturbances [18,19,20,21,22,23]. Regarding the lipid disorders, the role of triglyceride-rich lipoproteins (TRLs) and their remnants has come to the forefront in the past decade [24,25,26,27]. Triglycerides (TGs) stand as markers of the lipoproteins that contain them, and more specifically of the remnants of the catabolism of TRLs, which tend to contain twice as much cholesterol as compared to LDL. The accumulation of circulating TRLs and their partially lipolyzed derivatives, known as “remnants”, is caused by ineffective intravascular TG metabolism and recapture. These cholesterol-enriched remnant particles are hypothesized to contribute to atherogenesis in addition to LDL [8,24,27,28,29,30,31,32,33,34,35].

Even though epidemiological studies have long associated plasma TG levels with the risk of atherosclerotic cardiovascular disease, the association between TG levels and other risk factors has led many researchers, but not all, to hypothesize that the relationship is confounded and likely not causal. Certainly, TGs per se are neutral from the standpoint of pure chemistry and are also neutral from the viewpoint of a direct pathological effect on atherogenesis.

This opinion, however, completely changed when it was discovered that variations in TG levels caused by genetics, but not by low high-density lipoprotein cholesterol (HDL-C) levels, were linked to ASCVD [24,25,27,28,36]. Additionally, clinical outcome studies conducted to evaluate the advantages of raising HDL-C levels failed to detect a decrease in the risk of cardiovascular events. Therefore, management of increased TRLs has emerged as the next prospective lipid-lowering method to minimize ASCVD risk, although low HDL-C levels are now regarded as an indicator of ASCVD risk (mainly because they are a marker of TRL decreased turnover) but not a target for therapy [3].

Chylomicron and very low-density lipoprotein (VLDL) intravascular catabolism results in a variety of remnant particles that have undergone partial lipolysis. Lipases, lipid transfer proteins, and the number of exchangeable lipoproteins all have an impact on the amounts and characteristics of these molecules in plasma [27,37,38,39,40,41,42,43,44,45,46,47]. Remnants may develop pathologic characteristics that aid in the progression of ASCVD, such as elevated cholesterol levels and the transport of thrombogenic and inflammatory mediators, during their plasma transit [7].

In the past decade, a plethora of information has emerged on the role of TRL metabolism and their resulting remnant particles in atherogenesis, which has also sparked the development of new therapeutical targets.

The aim of the present narrative review is to briefly summarize the main pathways of TRL metabolism, bringing to the forefront the newly discovered role of apolipoproteins, the key physiological function of lipoprotein lipase and its main regulators, the importance of the fluxes of these particles in the post-prandial period, their catabolic rate and the role of apoC-III and angiopoietin-like proteins in the partition of TRLs during the fast-fed cycle. For the sake of brevity, we do not highlight here the role of HDLs, which is covered in several other reviews. Finally, we provide a succinct summary of the new and old therapeutic armamentarium and the outcomes of the many trials as well as those that are ongoing with a final outlook on different methodological approaches to measuring TRL remnants, still in search of the gold standard.

## 2. Origins of Circulating Triglyceride-Rich Lipoproteins

TGs provide an efficient and compact energy source. The development of metabolic processes to store and retain available TGs for usage in a controlled manner was required because humans have had relatively limited and sporadic access to food containing high quantities of fat for a large portion of their evolutionary history as hunter-gatherers. To effectively utilize this resource, highly regulated and efficient procedures are used to move TGs between sites of absorption (intestine), storage (adipose tissue), repackaging (liver), and usage (muscle). Circulating TRLs stem from the liver in the form of VLDLs carrying TGs produced in the body in an endogenous TRL pathway. TRLs coming from food are synthesized by the intestines in the form of chylomicrons [8,12,18,24,26,29,41]. Both pathways have similarities and differences that we will review in the following paragraphs.

### 2.1. Endogenous Pathways; VLDL Synthesis by the Liver Occurs throughout the Day and Is Critical during Fasting (Figure 1)

Triglycerides produced in the liver are transported by VLDL to the body’s peripheral tissues for utilization. These lipoproteins transport over 90% of the TGs in the bloodstream while fasting. Overall, the amount of fat transported in this way is over 50 g per day. These TGs come from fatty acids (FAs) from a variety of sources including chylomicron remnants, hepatic de novo lipogenesis (DNL, the process by which carbohydrates are transformed into FAs), uptake of non-esterified (free) fatty acids (FFAs) from the plasma, and release of FAs from hepatocyte cytosolic lipid droplets [1,24,30,40,41].

**Figure 1:**
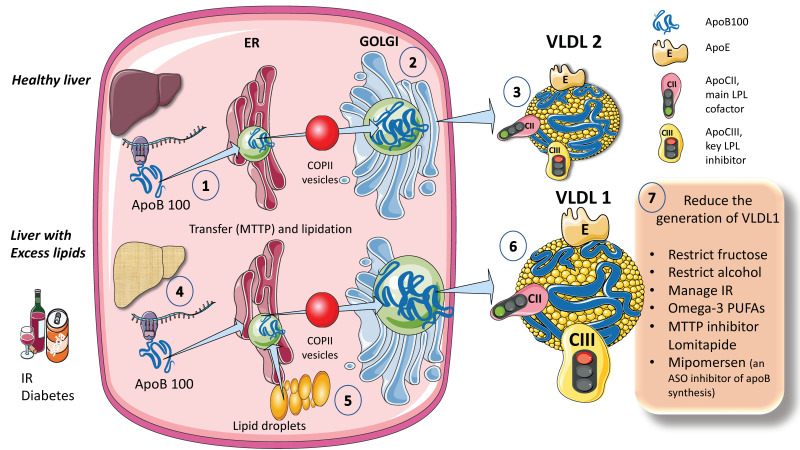
**Production of endogenous triglyceride-rich lipoproteins (TRLs) by the liver. A. Healthy liver** (1) ApoB100, one of the largest proteins, is synthesized by hepatocytes in the reticulum (ER) where it is moved along by the concourse of microsomal triglyceride transfer protein (MTTP). (2) Lipidated apo B is moved to the Golgi apparatus by means of COP II (Coat Protein Complex II) vesicles. (3) Finally, very low-density lipoprotein 2 (VLDL2), containing phospholipids and cholesterol besides TG, is secreted into the circulation. B. Liver with excess lipids. (4) TG employed for the synthesis of VLDL may come from re-esterified fatty acids catabolism of remnants or de novo lipogenesis (DNL). DNL implies the synthesis of fatty acids from glycerol and acetyl CoA derived from the metabolism of glucose and fructose. DNL is only 5% (of the total FA synthesis) in normal livers, but in (5) and (6) it reaches 25% when there is insulin resistance, diabetes, and high consumption of sugars or alcohol. Excess fat from DNL, build-up of circulating fatty acids, or liver steatosis is employed to enrich VLDL2 to a much larger particle called VLDL1. As compared to VLDL2, VLDL1 is not larger, but it contains more apoCIII. As we illustrate later, apoCIII is a very potent inhibitor of the catabolism of VLDL, thus promoting hypertriglyceridemia. The prolonged residence time favored by a delayed catabolism promotes the action of hepatic (HL) and endothelial lipase (EL) that ultimately results in the production of small and dense LDL, which is highly atherogenic. (7) We summarize some of the dietary and pharmacological molecules that reduce the production of VLDL1. Unfortunately, but not surprisingly, the pharmacological agents result in the accumulation of TG in the liver, thus producing a fatty liver. The figure was partly generated using Servier Medical Art, provided by Servier, licensed under a Creative Commons Attribution 3.0 unported license.

The process for assembling VLDL is intricate and strictly controlled. We provide a simplified diagram (Figure 1A). Microsomal triglyceride transfer protein (MTTP) mediates the co-translational lipidation of the developing apoB polypeptide in the endoplasmic reticulum (ER), which is the first step in this route and forms a pre-VLDL particle. Pre-VLDL can be further lipidated to generate VLDL2, a lipoprotein with a tiny, triglyceride-rich core when the chaperones separate and the lipidation is sufficient to enable the proper folding of apoB (Figure 1A). ApoB100 is one of the largest known proteins, with a mass of 500 kDa. In normal conditions, but much increased in livers with excess fat (Figure 1B) large triglyceride-rich VLDL1 is created by fusing nascent VLDL2 with lipid droplets in the Golgi apparatus. This VLDL1 is then released into the bloodstream as well. About 45,000 triglyceride molecules per particle make up VLDL1, while just 10,000 TG molecules per particle make up VLDL2 [24]. Their metabolism is slightly different, as we shall examine later.

When liver TG levels are high, the production of VLDL1 is accelerated, probably to shield hepatocytes from the harmful effects of TG overload (Figure 1B). VLDL1 synthesis is significantly influenced by insulin, a key regulator of metabolic processes related to energy storage and consumption (particularly, fat and carbohydrate metabolism). Insulin inhibits the release of VLDL1 from the liver both directly and indirectly (by reducing FFA flow into the liver). Direct inhibition of VLDL1 production occurs postprandially (when insulin levels are elevated). In people with insulin resistance (IR), elevated amounts of liver fat, and type 2 diabetes mellitus, both the direct and indirect effects of hormones are impaired [24,25,26,27,29]. Drinking alcohol causes a dose-dependent increase in hepatic FA production and a dose-dependent decrease in FA oxidation, which has the net effect of increasing VLDL secretion. The synthesis of VLDL2 is more tightly related to cholesterol metabolism and is not directly influenced by insulin. Endogenous cholesterol production is boosted when VLDL2 secretion is increased possibly to control hepatic cholesterol levels. VLDL2 rather than VLDL1 is generated in greater quantities in people with elevated plasma cholesterol levels, which may be a factor in the LDL-C overproduction that is frequently observed in these patients [8,27,34,35,42].

As shown in Figure 1, VLDL1 and VLDL2 contain several surface apolipoproteins other than apoB100, many of which are acquired in plasma, transferred from HDL. For the sake of our discussion the most important ones, which we illustrate in the figure, are apoE, which is critical for the binding of these particles to receptors for uptake, apoCII, the main activator of lipoprotein lipase, and apoCIII, the main inhibitor. We also illustrate in this figure that one main difference between VLDL1 and 2 is that VLDL1 contains more apoCIII [6,16,30]. We elaborate on the significance of these differences in a later section of this review.

### 2.2. Exogenous Pathways; Chylomicron Synthesis by Enterocytes Occurs in the Postprandial Period

**Figure 2:**
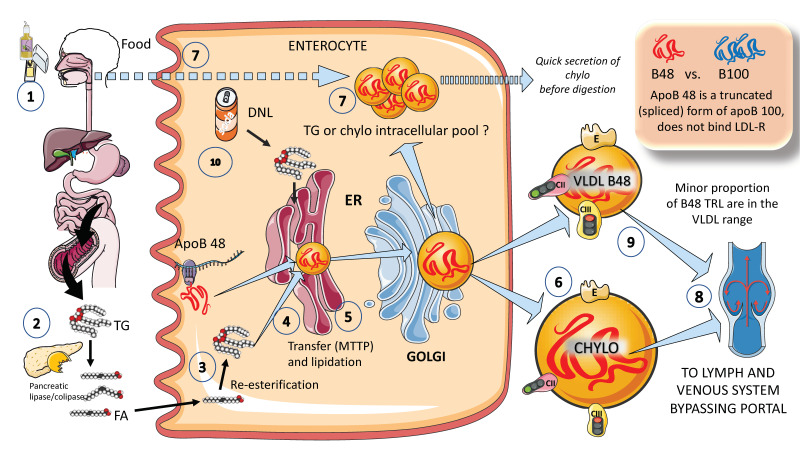
**Production of exogenous triglyceride-rich lipoproteins (TRLs) by the liver: Chylomicrons and VLDL apoB48.** (1) Lipids in the diet are (2) digested by pancreatic lipase, which is activated by liver co-lipase, in a complex system of micelles, resulting in the absorption of fatty acids and monoglycerides. (3) Fatty acids are re-esterified by the enterocyte and the resulting TGs (4) serve to lipidate apoB48. The latter is a truncated, spliced form of apoB100, which lacks the LDL receptor binding sites. (5) Much less is known about the transit and steps in chylomicron production as compared to VLDL. The resultant chylomicrons, which are very large particles, are (6) secreted into the circulation (7). However, very recently it has been shown that some of them are either stored as an intracellular pool or swiftly produced from TG droplets that can be called upon very quickly when the next meal comes, following signals stemming from the intake of more food and even before it reaches the intestines. (8) Chylomicrons secreted into the circulation contain phospholipids, cholesterol, and the liposoluble vitamins and are first transported by the lymph, reaching the venous, and finally the arterial circulation in a third step. (9) It has recently been shown that some of the apoB48-containing lipoproteins coming from the intestines are also in the VLDL size range. The figure was partly generated using Servier Medical Art, provided by Servier, licensed under a Creative Commons Attribution 3.0 unported license.

In Figure 2, we illustrate, in a simplified outline, the main features of chylomicron production by enterocytes. The chylomicron assembly pathway is substantially less well understood than the liver VLDL assembly pathways, and it transports all fat in the diet (over 70 g per day) with the exception of short-chain fatty acids. Several theories have been put forth to explain how these enormous TRLs form. As shown in the figure, a fat load in food is digested by pancreatic lipase with the activation provided by liver co-lipase in a complex system of micelles that ultimately leads to the production of free fatty acids and monoglycerides [39,40]. After absorption by the enterocyte, these fatty acids are re-esterified into TG, which represents the first point of regulation of the composition of these molecules, i.e., the relatively amount of saturated versus mono and poly and saturated fatty acids. The backbone of chylomicrons is apoB48, which represents a truncated, spliced form of apoB100 that is missing from the LDL receptor binding site. In a similar, but less well-described process, apoB48 is lipidated with TG, phospholipids, and cholesterol through its passage across the ER and Golgi apparatus [48,49,50,51,52]. Large triglyceride-rich chylomicrons and primordial, lipid-poor, apoB48-containing lipoproteins may assemble through separate mechanisms. As shown in Figure 2, chylomicrons that have just been secreted are discharged into lymphatic channels and go to the thoracic duct, where they are transported with their load of dietary fats and fat-soluble vitamins to the left subclavian vein. Therefore, most dietary lipids do not enter the hepatic portal system like other nutrients do [50,52].

Of note, contrary to what was previously believed, recent research has demonstrated that some TGs are retained and stored in the intestines, and that the first source of TGs produced after a meal may come from previous food intake [7,24,25]. This theory suggests that intestinal cells can start secreting stored TGs in chylomicrons immediately after meal consumption without the need to wait for dietary lipids to pass the enterocyte boundary. The taste-gut-brain axis has been connected to the release of chylomicrons, which may help to explain why fat or glucose need to be just tasted and not necessarily consumed to produce chylomicrons.

Finally, apoB 48-containing TRL is now known to be secreted both continuously at a low rate in the fasting state and at a greater rate postprandially. They are secreted not only as chylomicrons but also as smaller lipoproteins detected in the VLDL density range, as we depict in Figure 2 [24,29].

A parallelism exists between the liver and the intestine as to which factors affect the production of chylomicrons. Indeed, FFAs and insulin both control chylomicron synthesis and release. In people with insulin sensitivity, insulin inhibits the release of apoB48-containing lipoproteins from the intestine, but in insulin-resistant conditions, such as metabolic syndrome (MetS) or type 2 diabetes, this regulatory function is compromised, leading to an overproduction of chylomicrons and apoB48-VLDL. Additionally, it appears that incretins play a significant role in altering chylomicron secretion via receptor-mediated pathways and neuronal networks [24,26,29,39].

## 3. The Second Phase of the Fluxes of TRLs: Enabling the Uptake of Fatty Acids by Tissues—The Role of Lipoprotein Lipase

Given the frequency of hypertriglyceridemia in the general population, which amounts to more than 30%, one very important question we must answer is the cause. Is this due to either:

1. Excess production by the process we have just described?
2. Delayed second phase, delivery of free fatty acids to tissues (lipolysis) or;
3. Delayed uptake of the remaining molecules after lipolysis (remnants)?
4. All of the above?

In this regard, analysis of some quantitative data provides interesting insights. The normal ranges of apoB100 and triglyceride secretion, roughly three-fold and six-fold, respectively, cannot explain why plasma triglyceride levels in the population may span a range from 50 to 2000 mg/dL, despite the fact that enhanced VLDL secretion is an important role in the genesis of dyslipidemia [24,25,53]. The straightforward response is that the absolute rate of entry into plasma and the effectiveness of removal (fractional clearance rate, or FCR) determine plasma concentration. In the case of TRLs, the latter is established by the direct removal of remnant VLDL and chylomicron particles (along with the remaining triglyceride they carry) from the circulation and lipolysis of the triglyceride in VLDL and chylomicrons. In order to understand the quantitative component of each one of the processes, it is imperative to review the physiology of lipoprotein lipase as well as the biology of TRL remnants and the processes that determine their uptake and removal from the circulation.

### 3.1. Lipoprotein Lipase (LPL) and Other Lipases

Post-heparin lipases are a group of enzymes that includes LPL (lipoprotein lipase), HL (hepatic triglyceride lipase), and EL (endothelial lipase) and work to remove TGs from VLDL and chylomicrons. LPL is mainly active in larger TRLs, whereas HL and EL prefer smaller VLDL particles, remnants, IDL (intermediate-density lipoproteins), and HDL. These enzymes have different preferred substrates. Fasting hyperchylomicronemia with severe hypertriglyceridemia results from a total loss of LPL, which is required for the initial conversion of TRL-TGs into fatty acids. In non-hepatic tissues, lipolysis of chylomicrons typically eliminates between 50% and 70% of the core triglyceride and breaks down larger chylomicron into smaller chylomicron remnant particles [54,55,56,57].

#### 3.1.1. LPL

**Figure 3:**
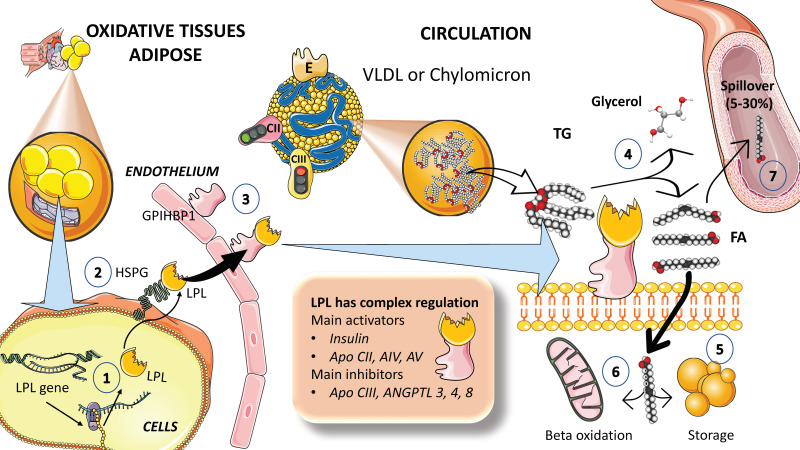
**Lipoprotein lipase is the key controller of TRL fluxes.** Current data support the contention that delayed turnover or catabolism of TRL is more important than excess production in the pathophysiology of hypertriglyceridemia. (1) Lipoprotein lipase is synthesized by the cells in oxidative tissues such as muscle (skeletal and myocardium) as well as adipocytes and the mammary gland, among others. (2) LPL is an extremely complex enzyme that requires heparan sulfate proteoglycans (HSPG) on the surface of the cells to help in the transcytosis of the molecule to the luminal face of the capillaries. (3) Glycosylphosphatidylinositol anchored high-density lipoprotein binding protein 1 (GPIHBP1) serves to anchor LPL on the luminal surface of the endothelial cells and aids in the conformation of LPL to an active lipolytic enzyme. (4) LPL acts on TRLs to hydrolyze TGs into glycerol and free fatty acids. Free fatty acids are employed for storage in adipocytes, as shown in (5), or for oxidation in muscle and myocardial tissue, as shown in (6). (7) Some of the fatty acids (that may amount to 5 to 30%) remain in the circulation and are referred to as *spill over* fatty acids. LPL has a very complex regulation: its main activators are insulin, apoCIII, AIV, and AV (see below), and its main inhibitors are apoCIII, and angiopoietin-like protein (ANGPTL) 3, 4, and 8. The figure was partly generated using Servier Medical Art, provided by Servier, licensed under a Creative Commons Attribution 3.0 unported license.

Survival depends on strict triglyceride transport control. TGs are saved for use while they are in abundance, and when they are needed for energy production, they are released from fat depots and sent in the right direction. Crucial regulatory elements have been found in the past decade that provide a better understanding of TRL metabolism. The primary enzyme in the intravascular breakdown of TGs in circulating TRLs is lipoprotein lipase (LPL), which catalyzes the release of FAs for storage by adipose tissue or usage as an energy source by muscle (Figure 3). LPL is generated in macrophages, adipose tissue, skeletal and cardiac muscle, and the brain, but not in the liver. LPL works intravascularly, precisely at the luminal surface of the endothelium. Multiple modulators work together to control the intricate regulation of LPL synthesis and activity [54,55,56,58,59,60]. For instance, insulin appears to control LPL activity in adipocytes at both the post-transcriptional and post-translational levels (Figure 3).

To serve as a gatekeeper for local FA absorption, LPL expression is regulated tissue specifically. LPL, following production and maturation in the ER, is delivered from cells (adipocytes, skeletal, cardiac myocytes, etc.) to the endothelial surface of capillaries where it is activated. The ER plays a critical role in processing nascent LPL, and several important regulators are involved in this process. LPL binds to heparin sulfate proteoglycans (HSPG) on the surface of the cells to help in the transcytosis of the molecule to the luminal face of the capillaries [55,58,60,61,62]. Lipase maturation factor 1 (LMF1) and glycosylphosphatidylinositol-anchored HDL-binding protein 1 (GPIHBP1) act as chaperones for LPL from its site of synthesis to the endothelial surface and help anchor the enzyme to endothelial cells in capillaries, respectively (Figure 3). Prior research suggested that LPL could only be active as a homodimer; however, further investigations suggest that LPL can also be active when it forms a complex with GPIHBP1 [54,55,61,63].

**Figure 4:**
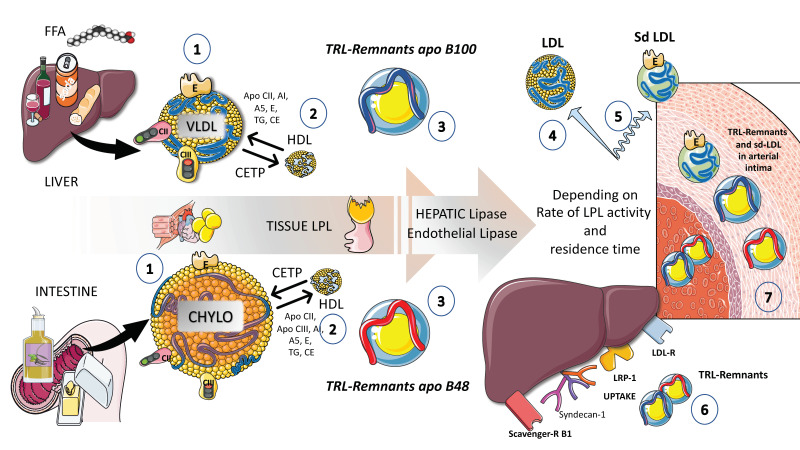
**Putting together TRL fluxes: general outline of endogenous and exogenous TRL turnover.** (1) Endogenous TRLs coming from the liver as well as, in the postprandial phase, exogenous TRLs coming from the intestines, are protagonists of a series of complex reactions and transfers of proteins and lipids in the circulation. The main physiological pathway is to deliver fatty acids to the tissues in need in a regulated fashion. Both TRL species undergo lipolysis by lipoprotein lipase, but chylomicrons are the preferred substrate. It must be emphasized that VLDL production occurs throughout the day. Therefore, an excess of chylomicrons may result in the accumulation of VLDLs. (2) Under the action of LPL, VLDL and chylomicrons are considerably reduced in size and the excess phospholipids and cholesterol are transferred to HDL. HDL plays a key role in the metabolism of TRL because it also provides many of the surface apolipoproteins that are significant actors in this process. Cholesterol-ester transfer protein (CETP) plays a very important role in the transfer of esterified cholesterol from HDLs from VLDLs and chylomicron particles as well as receiving TGs from these molecules, which passes to HDLs. (3) The resulting partially delipidated molecules become enriched in cholesterol esters and are called remnants. (4) In the case of VLDLs, an intermediate remnant is called an IDL or intermediate density lipoprotein that ultimately is delipidated into LDL, which loses the surface apolipoproteins and contains only apoB100 and apoE. (5) When this delicate process is perturbed and there are delays in intravascular catabolism, some of the remnants are substrates for very active hepatic and endothelial lipases and the final product is small-dense LDL, which is far more atherogenic than large buoyant LDL. (6) TRL remnants are taken up by the liver via several receptors, as we show in the figure: LDL receptor, LDL receptor like-protein1, scavenger receptor B1, and syndecan 1. This is the pathway by which dietary liposoluble vitamins and cholesterol finally reach the liver, in the form of chylomicron remnants. (7) Delays in any part of this process result in the transfer of the TRL remnants into the arterial intima. Remnants are less abundant but contain twice as much cholesterol esters as LDL and are equally or more atherogenic. The figure was partly generated using Servier Medical Art, provided by Servier, licensed under a Creative Commons Attribution 3.0 unported license.

Insulin responses, together with the moderating effects of other hormones and proteins that regulate required shifts in the lipolytic rates between fasting and postprandial states, govern the rapid adaptive changes of LPL to dietary changes (Figure 3). TRL metabolism is impacted in diverse ways by numerous apolipoproteins found on their surface. Depending on the nutritional or metabolic condition, apoCI, apoCII, apoCIII, and apo E are transferred between TRL and HDL particles but not apoB [55,64]. According to studies, apoCII and apoAV increase LPL activity, whereas apoCI, apoCIII, and apoE decrease LPL activity (Figure 4). ApoCII appears to be a rate-limiting component in lipolysis that is mediated by LPL. A recently synthesized CII-mimetic peptide is a potent activator of human LPL and may have triglyceride-lowering potential [54,63]. This evidence led to the discovery that a dual apoCII-mimetic/apoCIII-antagonist peptide can lower plasma triglyceride levels. Development of these agents is still in preliminary phases [64].

#### 3.1.2. Overview of the Main Events Governing TRL Metabolism in the Postprandial Phase

Before studying the intricacies and fine-tuning of the regulation of the activity of lipoprotein lipase, let us take a bird’s eye view of the main processes that occur intravascularly with regard to the metabolism of chylomicrons and VLDL.

Acquiring exchangeable apolipoproteins (apos) from HDL occurs concurrently with the entry of chylomicron and VLDL particles into plasma from the intestine and liver, respectively (Figure 4).

These include apoCII, which triggers lipoprotein lipase to initiate triglyceride hydrolysis, apoAV, which encourages this process, as we will discuss later, and others, most notably apoCIII and apoCI, which can hamper it. ApoE, apoAI, and apoAII are additional proteins that can be obtained from HDL [12,39,40,65,66]. After lipolysis begins, as shown in Figure 4, some exchangeable apos are shed to HDL, and exchanges of surface and core lipids with HDL and low-density lipoproteins (LDLs) are mediated by phospholipid and cholesteryl ester transfer proteins (CETPs), respectively. Precisely, this process explains the frequent association of hypertriglyceridemia with low HDL cholesterol levels, since CETP transfers TGs to the HDL and cholesterol to the remnant or LDL particle. This is why low HDL cholesterol is in fact a marker of TRL dyslipidemia and it is not pathogenic per se. It is a surrogate indicator of the presence of remnants in the circulation that are indeed atherogenic. The role of HDL in these processes cannot be overemphasized but it will not be discussed in detail in this review. The reader is referred to reviews on the matter [41,67,68].

Together, these activities produce particles known as remnants, with the primary structural proteins of chylomicron and VLDL remnants, respectively, being retained by apoB48 and apoB100. In the case of VLDL remnants, these consist of a group of particles known as intermediate-density lipoproteins (IDLs), with a density (d) of 1.006–1.019 g/mL and an analytical ultracentrifuge flotation rate (Sf) of 12–20, which has been demonstrated to overlap in distribution with small VLDL particles with d 1.006 g/mL and Sf 20–60 [7,12,37,38,39,69].

Although there are only a few unmodified nascent VLDLs and chylomicrons present at any given time due to LPL quick activity, all particles in this density range (d 1.006 g/mL) contribute to the remnant spectrum to variable degrees. This complexity underscores the difficulties currently found to measure remnant particles accurately, as we will discuss later.

Remnant particles are hydrolyzed further by hepatic triglyceride lipase (HL), during passage through the hepatic sinusoids, and gain additional apoE, which facilitates their binding and uptake by proteins on the surface of the liver cells, such as the LDL receptor (LDLR), LDL-like receptor protein-1 (LRP-1), and the heparin sulfate proteoglycan syndecan-1 (Figure 4). Additionally, their apoAV content encourages receptor-mediated remnant absorption, as will be detailed later. The larger size of chylomicron remnants and the lesser surface area of apoB48 vs. apoB100 when compared to VLDL allows chylomicron remnants binding of more apoE and, thus, higher hepatic absorption and shorter plasma residence time under normal metabolic circumstances [27,30,70,71,72]. Plasma chylomicron metabolic products typically remain relatively large, lipid-rich, and buoyant, but as VLDL remnants are gradually broken down, IDL particles are created, which then advance to LDL (Figure 4) [29]. A recent elegant kinetic study with radioisotopes in humans has shown that there is a flux of 50 plasma pools of chylomicrons per day. If we compare with the fluxes of VLDLs, these are much slower: those are 10 pools per day for VLDL1, 3.8 pools per day for VLDL2, and 2 pools per day for IDL [29].

**Figure 5:**
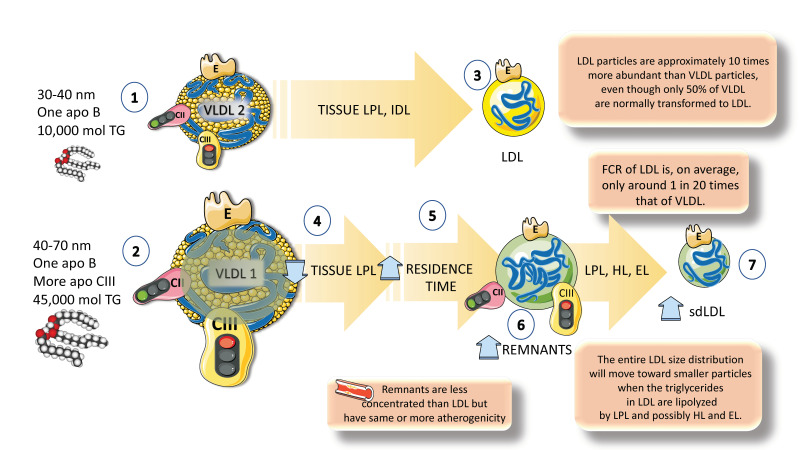
**Another layer of complexity: differences between VLDL1 and VLDL2 catabolism.** (1). VLDL2 is a particle of 30 to 40 nanometers and contains 10,000 moles of TGs. (2) VLDL1 instead, is twice as large (40 to 70 nanometers) and contains 45,000 moles of TGs. (3) VLDL2 tends to follow a more regulated and faster pathway leading to the production of LDL particles. Turnover of LDL is 20 times slower than that of VLDL and that is the reason why LDL particles are approximately 10 times more abundant than VLDL particles. Moreover, only 50% of VLDLs are normally transformed into LDLs, the rest are intermediate-density LDLs (IDLs) and other remnants. (4) VLDL1 contains far more apoCIII, which is the main inhibitor of lipoprotein lipase. Consequently, its catabolism tends to be slower, with an increased residence time. (5, 6) The accumulation of more remnants creates opportunities for the interaction with hepatic and endothelial lipase, which leads to the production of (7) smaller LDL particles, which are more atherogenic than large, buoyant LDLs. The figure was partly generated using Servier Medical Art, provided by Servier, licensed under a Creative Commons Attribution 3.0 unported license.

As shown in Figure 4 and Figure 5, the breakdown of VLDLs produces smaller particles known as IDL or VLDL remnants. Although the liver essentially absorbs all chylomicron remnants, VLDL and IDL remnants can either be eliminated by the liver or further lipolyzed into LDLs, most likely by the actions of hepatic lipase (HL). The percentage of VLDLs converted to LDLs in human studies using radioactive iodine or stable isotopes of amino acids ranges from 25% to 75%; the determinants of this wide range of conversion and, conversely, of hepatic removal of VLDL remnants and IDLs, are unknown, but HL and EL are likely involved [10,33,34]. Lower conversion rates of TRLs to LDLs are observed in people with greater plasma levels of TRLs. LDL particles are approximately 10 times more abundant than VLDL particles, even if only 50% of VLDLs are normally transformed into LDLs. This is because the FCR of LDLs is, on average, only around 5% of that of VLDLs.

In people with moderate hypertriglyceridemia, the LDL FCR is normal, but it can rise in those with severe hypertriglyceridemia. In addition to influencing LDL particle counts, elevated TRLs and TRL remnant levels also significantly impact LDL particle size and composition and are associated with increased levels of small-dense LDL. Triglyceride-rich LDL is produced by increased VLDL production, ineffective lipolysis of TRL TGs, and/or decreased hepatic uptake of TRL remnants [10]. The entire LDL size distribution will move toward smaller particles when the TGs in LDL are lipolyzed by LPL and possibly HL and EL (Figure 4 and Figure 5).

The mechanisms governing the size distribution and cholesterol content of LDL are the same as those causing the well-known inverse correlation between HDL and triglyceride levels discussed above. The cholesterol-ester acyl transferase (CETP) exchange of TRL-TG for HDL-CE when TRL levels are high, decreases HDL-C (HDL cholesterol) and produces substrates for HL and EL, which in turn reduces the overall size and lipid content of HDLs as well. Smaller HDLs are removed from the blood more quickly than larger HDLs, which are both cleared by the kidney and liver. Deficient LPL activity also leads to a decrease in the production of lipids and proteins from TRLs, which typically contribute to HDL size and quantity [67]. 

## 4. Apo CIII: A Major Inhibitor of LPL Activity with Physiological and Pharmacological Importance

**Figure 6:**
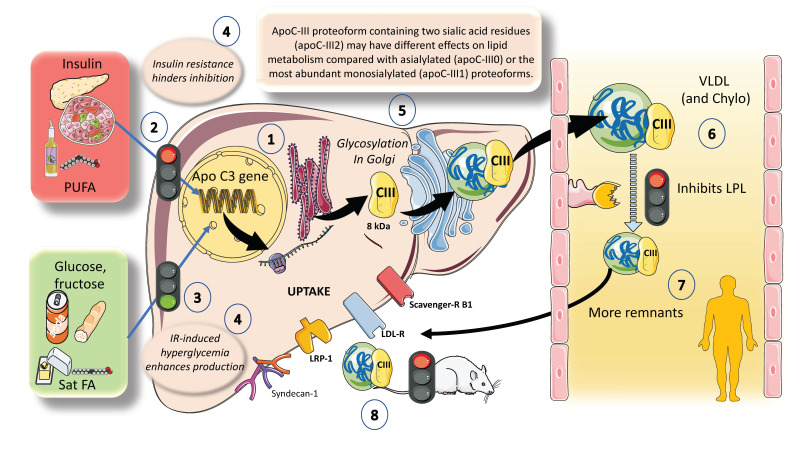
**ApoCIII: a major inhibitor of LPL activity with physiological and pharmacological importance.** (1). Hepatocytes have the apoCIII gene for a protein of about 8 kilodaltons. Its regulation is complex but can be summarized as follows: (2) The two main inhibitors are insulin and polyunsaturated fatty acids, whereas (3) the main enhancers are glucose, fructose, and saturated fatty acids. (4) In this regard, insulin resistance (IR) negates the inhibition promoting increased expression of an ApoCIII delayed catabolism of TRLs, as seen in metabolic syndrome and diabetes, and at the same time produces hyperglycemia, which directly stimulates the production. Diets very rich in sugar or saturated fat enhance the production of apo CIII. (5) ApoCIII is a small protein that gets glycosylated in the Golgi apparatus and circulates in three isoforms with 0, 1 (most abundant), or 2 sialic acid residues. Isoform distribution may have a bearing in the final activity of the protein. (6) ApoCIII in VLDL and chylomicrons (which get it by transfer from VLDL or from HDL) potently inhibits lipoprotein lipase activity and acts as a counterpart of the main activator, which is ApoCII. Other key inhibitors are apoAV, ANGPTL3-4 and 8, as we show later. (7) Excess activity of apoCIII result in increased residence time of VLDLs and chylomicron remnants. (8) As indicated earlier, remnants are taken up by several receptor mechanisms in the liver. ApoCIII also inhibits this reuptake, especially in rodents. The activity in humans has lately been regarded as less important when compared to its action on LPL. The key role of apoCIII in this process as well as results from animal and human loss of function studies have uncovered the potential role of apoCIII inhibitors as a therapeutic avenue for hypertriglyceridemia, as we further discuss in this review. The figure was partly generated using Servier Medical Art, provided by Servier, licensed under a Creative Commons Attribution 3.0 unported license.

ApoCIII has been causally linked to an increased risk of ASCVD and is a key regulator of plasma triglyceride and TRL levels. Although ApoCIII inhibits LPL activity, its proatherogenic effects also appear to be influenced by LPL-independent mechanisms [73,74]. As shown in Figure 6, hepatocytes have the ApoCIII gene for a protein of about 8 kDa. Its regulation is complex but can be summarized as follows: the two main inhibitors are insulin and polyunsaturated fatty acids, whereas the main enhancers are glucose, fructose, and saturated fatty acids. ChREBP and other factors mediate glucose stimulatory effects. Both in vivo and in vitro studies have shown that insulin inhibits ApoC-III production through the forkhead box protein O1 [24,27,28,29,41,75]. 

The deregulation of apoCIII metabolism observed in people with diabetes appears to be the logical result of the combined actions of insulin and glucose. Additionally, saturated FA stimulate and polyunsaturated FA (PUFA) inhibit ApoCIII expression. In this regard, insulin resistance negates the inhibition promoting increased expression of apoCIII followed by a delayed catabolism of TRLs, as seen in MetS and diabetes, and at the same time produces hyperglycemia, which directly stimulates the production of apoCIII (Figure 6). Independent of this, diets very rich in sugar or saturated fat enhance the production of apoCIII [45,46,75,76]. ApoCIII is a small protein that gets glycosylated in the Golgi apparatus and circulates in three isoforms with 0, 1 (most abundant), or 2 sialic acid residues, as shown in the figure. Isoform distribution may have a bearing on the final activity of the protein. ApoCIII in VLDL and chylomicrons (which get it by transfer from VLDL or from HDL) potently inhibits lipoprotein lipase activity and acts as a counterpart of the main activator, which is ApoCII. Other key inhibitors are apoAV and ANGPTL3-4 and 8, as we show later. Excess activity of apoCIII results in increased residence time of VLDL and chylomicron remnants [10,77]. As shown before, remnants are taken up by several receptor mechanisms in the liver. ApoCIII also inhibits this reuptake, especially in rodents [25,28]. Human studies support a major role for apoCIII as an inhibitor of LPL-mediated lipolysis, while mouse data support a chief role for apoCIII as an inhibitor of the removal of remnants of VLDL and chylomicrons by the liver, with minimal effect of LOF of apoCIII on hepatic remnant removal, except when LPL activity is significantly reduced or completely absent. Dietary intervention by reducing fructose consumption is a potent reducer of apoCIII, as we have recently shown [46,47]. The key role of apoCIII as an LPL inhibitor, as well as results from animal and human loss of function studies, have also uncovered the potential role of apoCIII inhibitors as a therapeutic avenue for hypertriglyceridemia, as we further discuss in this review.

## 5. The Axis Angiopoietin-like (ANGPTL) 3, 4, and 8 Controls the Partition of TRL Fluxes during Fasting and Feeding Cycles

**Figure 7:**
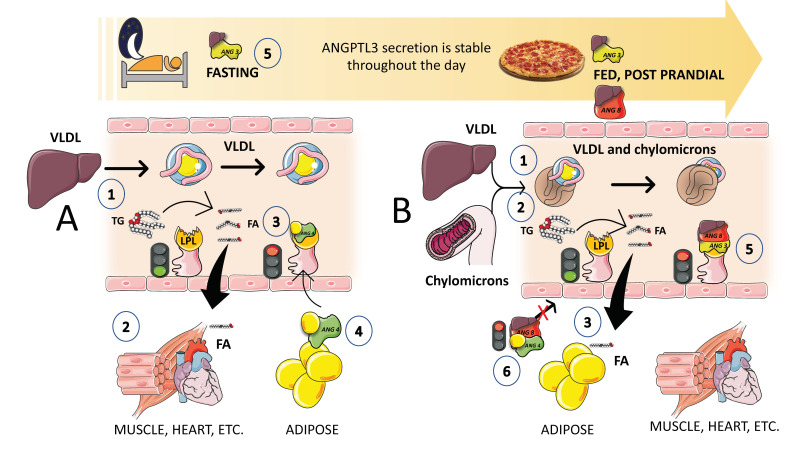
**The axis ANGPTL 3, 4, and 8 control the partition of TRL fluxes during fasting and feeding cycles.** Superimposed on the previously discussed regulation of LPL activity, there exists a finer control of the enzymes in different tissues that provides for the physiological partition of the TRL load depending on the needs of the body. Basically, during fasting, lipids are preferentially taken up by oxidative tissues such as cardiac and skeletal muscle, and storage at the adipocytes is not favored. On the other hand, upon feeding, LPL activity in adipocytes is much higher, and at the same time, its activity is reduced in oxidative tissues. In a nutshell, ANGPTL3 (secreted all day long) from the liver acts in an endocrine way to inhibit lipoprotein lipase in muscle and heart during the postprandial period. Conversely, ANGPTL4 secreted by the adipocyte acts in a paracrine fashion to inhibit lipoprotein lipase in adipose tissue during fasting. **A**. When fasting (1) VLDL secreted by the liver is preferentially hydrolyzed by (2) muscle and heart because adipose tissue LPL is inhibited (3 and 4) by the secretion of ANGPTL4, during fasting and cold situations. **B**. During the fed or postprandial phase, 1),2) hepatic VLDL and intestinal chylomicrons compete for the hydrolysis by LPL and this hydrolysis occurs preferentially in adipose tissue capillaries (3) because fasting promotes liver secretion of ANGPTL8 4, which strongly enhances the inhibitory action of ANGPTL3 on muscle and the heart. (5) The complex ANGPTL3-8 is far more active than ANGPTL3 alone. Fine regulation of the cycle depends on the postprandial liver secretion of ANGPTL8, which also removes the inhibition of ANGPTL4 on adipose tissue LPL (6). The result is that after a meal fat is preferentially partitioned to adipose tissue for storage. Note that this regulation is a fine-tuning of the regulations provided by insulin and the rate of apoCII and apoCIII on TRLs. The key role of ANGPTL3 in this process as well as results from animal and human loss of function studies have uncovered the potential role of ANGPTL3 inhibitors as a therapeutic avenue for hypertriglyceridemia, as we further discuss in this review. The figure was partly generated using Servier Medical Art, provided by Servier, licensed under a Creative Commons Attribution 3.0 unported license.

Superimposed on the previously discussed regulation of LPL activity, there exists a finer control of LPL activity in different tissues that facilitates the physiological partition of the TRL load depending on the needs of the body. Basically, during fasting, lipids are preferentially taken up by oxidative tissues such as cardiac and skeletal muscle, and storage at the adipocytes is not favored. On the other hand, upon feeding, LPL activity in adipocytes is much higher, and at the same time, its activity is reduced in oxidative tissues. It is now obvious that LPL plays a crucial role in TG trafficking and partitioning. LPL activity increases in white adipose tissue (WAT) after eating but decreases in muscles. On the other hand, during fasting, LPL activity decreases in WAT but increases in muscles. However, a significant portion of the mechanism governing tissue-specific LPL activity throughout the fed-fast cycle is yet unclear, although, as we shall see, considerable progress has been made in the past few years. LPL activity needs to be regulated at the tissue level to react to continually shifting metabolic conditions. Significant tissue-specific regulators of lipolysis, ANGPTL3, ANGPTL4, and ANGPTL8 have been identified (Figure 7). In a nutshell, ANGPTL3 (secreted all day long from the liver) acts in an endocrine way to inhibit lipoprotein lipase in muscle and the heart during the post-prandial period [78,79,80,81,82]. Conversely, ANGPTL4 secreted by the adipocyte acts in a paracrine fashion to inhibit lipoprotein lipase in adipose tissue during fasting (Figure 7).

Given that both ANGPTL4 and ANGPTL3 are effective LPL inhibitors, their discovery has significantly advanced our understanding of this process. In accordance with the most current hypothesis, ANGPTL8 stimulates ANGPTL3 in an endocrine manner to limit LPL activity in the heart and skeletal muscle, while ANGPT4 inhibits LPL activity in WAT by engaging intracellular and circulating species (Figure 7). Fasting increases ANGPTL4 but decreases ANGPTL8, which causes LPL activity in WAT to decrease and conversely to increase in muscles [77,83]. As a result, TGs are diverted to the muscles for oxidation. In contrast, eating decreases ANGPTL4 but increases ANGPTL8, increasing LPL activity in the WAT while decreasing it in the muscles, which directs the circulation of TGs to the WAT for storage (Figure 7). Since ANGPTL8 and ANGPTL3 are both secreted into the bloodstream by the liver and are not expressed in the heart or skeletal muscle, they are most likely to function in an endocrinal manner [79,80].

Insulin enhances the secretion of ANGPTL3 and ANGPTL8, and ANGPTL8 is elevated during eating. As shown in Figure 7B, in the postprandial state (when insulin levels are high), the presence of ANGPTL3 and ANGPTL8 in muscle decreases LPL activity and promotes TRL-triglyceride flow to adipose tissue. Endothelial lipase levels have also been shown to be suppressed by ANGPTL3 [83,84,85]. The key role of ANGPTL3 in this process, as well as results from animal and human loss of function studies, have uncovered the potential role of ANGPTL3 inhibitors as a therapeutic avenue for hypertriglyceridemia, as we further discuss in this review. Moreover, we have shown that higher ANGPTL3 can be found as early as in adolescents [72,77] with MetS and that, in an ongoing investigation, fructose restriction reduces their fasting and postprandial ANGPTL3. A better knowledge of the regulation of LPL has been achieved in the past few years with the discovery of the action and mechanisms of ANGPTL proteins together with apoAV (see below) being a strong activator of LPL.

### 5.1. ANGPTL8: A New Thrifty Gene?

The importance of LPL in the whole energy economy of the body has encouraged some speculation as to the role of these regulators in evolution. Indeed, according to the theory of evolution, inadequate calorie intake posed a serious hazard to survival during the evolution of humans. The thrifty gene theory postulates that individuals with these genes survived famines by accumulating more fat, and as a result, evolutionary selection favored genes and genetic variants that led to the buildup of adipose storage. Because one of its primary activities is to promote fat storage after a meal, ANGPTL8 might be considered a thrifty gene [78,80,81,86]. Constant feeding raises circulating ANGPTL8 levels, which results in increased adipose storage (obesity) and hypertriglyceridemia, and the same ANGPTL8 protein that likely protected human ancestors from starvation now predisposes people to metabolic syndrome.

The above suggests that it may be possible to reverse thrifty traits, such as obesity, circulating TG levels, and metabolic syndrome, by inhibiting ANGPTL8. In fact, ANGPTL8 deficiency in mice reduces TG levels in the serum and obesity. Injection of ANGPTL8 Ab in mice consistently decreased adiposity and circulating TG levels [80]. The future will tell whether this is a feasible new approach to curb this aspect of metabolic syndrome in humans.

### 5.2. It Is All about the Fluxes: Fractional Catabolic Rates and Residence Times

The final integration of the ANGPTL axis regulation of LPL with that provided by ApoCII and apoCIII remains to be elucidated. As has become clear in the discussion above, considerable progress has been made in the description of these two pathways, but we are still missing many links that in the future will allow for a better understanding of the process in a holistic manner. For instance, if we continue with an evolutionary viewpoint for these processes, access to fatty foods in our evolutionary past was episodic and needed a sustained energy expenditure (hunting). LPL seems to be a high-capacity, very effective enzyme for removing TGs from the center of TRL particles, and it is likely that an inhibitor of TRL catabolism works to assure slow distribution to several tissue sites rather than having the entire TG load in a chylomicron or VLDL1 particle removed at “first pass”, thereby depriving the rest of the body for a much-needed sustenance [24,25,28]. Thus, ApoCIII loss-of-function variants are uncommon in populations, which may be explained by the need for a prolonged circulation time for TRLs when dietary fat is scarce (as it has been for a large portion of human history). This circulation time is on the order of 30 min for chylomicrons and close to 12 h for VLDL, compared to less than 8 min and 3 h, respectively, when apoCIII is very low. It seems that nature favored or preferred a constant state of inhibition of LPL in order to keep a circulating mass of TG available before the following meal and for the longest possible time [24,25,28].

In a recent study, the physiological context of postprandial lipemia was used to study a group of unrelated patients with apoCIII LOF mutations [29]. The finding that direct clearance of VLDL was higher when apoCIII was low, suggests that remnant removal may be improved, and the observation that lowering apoCIII levels was associated with a rapid transit of TRL particles down the delipidation pathway suggests that the rate of remnant formation will be reduced. The production rates of apoB48- or apoB100-containing lipoproteins were unaffected, which is a significant finding showing that TGs exported from the liver and intestine may not be impaired using lipid-lowering drugs that target apoCIII.

## 6. Apo AV a Unique Modulator of Fasting and Postprandial TGs: The New Frontier?

**Figure 8:**
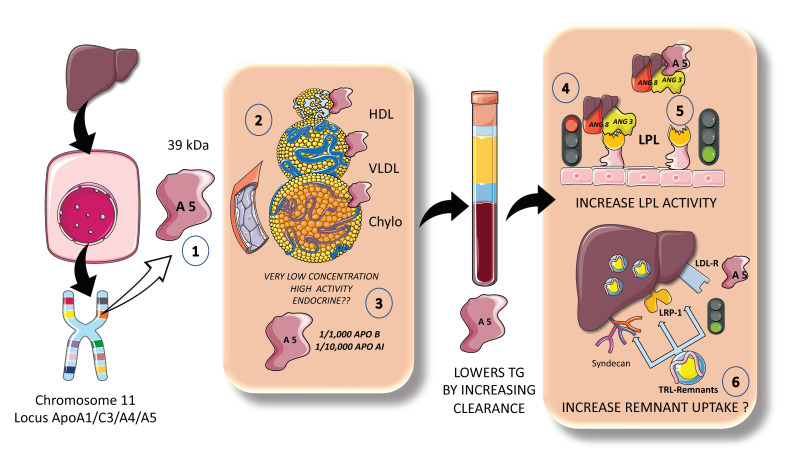
**Apo AV a unique modulator of fasting and postprandial TGs: the new frontier?** The locus ApoA1/C3/AIV on chromosome 11 has shown singular importance in lipid metabolism. In the past two decades, a new apo A, apoAV has been discovered and intensively studied. (1) ApoAV is 39 kDa protein synthesized exclusively by the liver which (2) circulates in HDL, VLDL, and chylomicrons but not in LDL. It circulates in extremely low concentrations but has proven to have strong activity as an LPL activator. (3) Indeed, its concentration is 1000-fold lower than that of apoB100 and 10,000 lower than that of apoAI. (4) Apo AV disrupts the inhibitory effect of the complex ANGPTL3-8, thereby (5) activating LPL. Some recent data indicate that it might also increase liver remnant uptake and removal. The figure was partly generated using Servier Medical Art, provided by Servier, licensed under a Creative Commons Attribution 3.0 unported license.

ApoAV is a particularly special lipid-modulating protein that is primarily synthesized in the liver but has effects in the gut, circulation, liver, and adipose tissue. On the human chromosome 11q23, the apolipoprotein gene cluster APOA1/C3/A4 was previously known to affect lipid metabolism [87,88]. Apolipoprotein AV was identified to be the newest member of this gene cluster to modulate plasma TGs in both mice and humans. Two separate teams independently identified ApoAV in 2001. Since its discovery, ApoAV has been called “a potent TG reducer” and has been characterized as having a “low concentration, high impact” (Figure 8). It seems that apoAV is expressed mostly in the liver [88,89]. The circulating, mature version of the protein is extremely hydrophobic, highly helical, and is largely bound to HDL and to a lesser extent to VLDL in rats. In human plasma, apoAV is found to be associated with HDL, VLDL, and chylomicrons, but not LDL (Figure 8) Heparin, heparin sulfate proteoglycans (HSPGs), and glycosylphosphatidylinositol high-density lipoprotein binding protein 1 (GPIHBP1) are all engaged by a group of positively charged amino acids found in apoAV [89]. Its plasma concentration in humans is extremely small when compared to other apolipoproteins, ranging from 20 to 500 ng/mL, which, when calculated on a molar basis, is around 1000 times lower than apoB100 and 10,000 times lower than apoA1 [89]. 

Given its extremely low circulating levels and distinct features, ApoAV has been found to be significantly different from other apolipoproteins. Some authors suggest that ApoAV operates more like a hormone, such as the incretins, and may be recycled by enterohepatic circulation. Moreover, small levels of apoAV released into bile may have a significant impact on the production and secretion of chylomicrons in the small intestine. According to preliminary research, apoAV is taken up intact by enterocytes while escaping luminal proteolysis, a rather unusual property [88,89].

Population-based studies collectively imply that apoAV polymorphisms that vary the amount of apoAV protein or the structure or function of the protein do affect lipid metabolism critically, potentially increasing the risk of developing cardiometabolic disease. Very modest amounts of apoAV can have a significant impact on plasma TG levels [89]. Numerous studies endorse the idea that apoAV plays a significant role in improving the clearance of plasma lipoproteins through improved TRL lipolysis and improved liver uptake of remnant particles (Figure 8). Will it become a pharmacological target?

## 7. The Postprandial Period, Remnants, and Atherogenesis: Time to Pay More Attention?

More than four decades have elapsed since Zilversmit, after his seminal work on the matter, proposed in 1979 that atherosclerosis is a postprandial phenomenon [65]. Analytical challenges, however, have determined that most of our diagnostic and therapeutic targets only take into account the fasting lipoprotein and lipid values. It must be remembered that we spend most of the day in the postprandial and more research is needed to account for this fact. Now that we have reviewed the main regulatory steps in the management of TG loads in the body, it is a good time to put these concepts together and further analyze the importance of the postprandial period and the many aspects of it that deserve more investigation. Chylomicrons and VLDL containing apoB-48 make up the wave of TRLs that emerges in the postprandial state. These TRLs compete with hepatic TRLs in the lipolytic and remnant elimination pathways (Figure 4 and Figure 5), representing a dynamic ‘load’ in addition to VLDL that is issued nearly continuously throughout the day. Although dietary fat in chylomicrons accounts for around 80% of the increase in plasma triglyceride levels after a meal, increases in apoB-48 and apoB-100—containing VLDL—are what cause the increase in particle number [40].

The competition for available LPL, for which large chylomicrons are the preferred substrate, may be the cause of postprandial increases in circulating VLDL1. This competition is what explains why fasting plasma triglyceride levels are closely correlated with the amount of alimentary lipemia that results from ingesting fat [1,6,7]. Additionally, not all FAs produced from chylomicrons by LPL are absorbed into tissues, rather, some are washed into the circulation as FFAs. This process is known as spillover. It is now understood that this contribution of dietary FAs—roughly 5–30% of FA to the plasma FFA pool after a meal—is a significant occurrence in the postprandial period. Therefore, dietary FA spillover may possibly be a factor in the postprandial increase in circulating liver-derived VLDL, as these FAs are re-esterified and packaged as VLDL-TGs [18,19,20,24,25,28].

**Figure 9:**
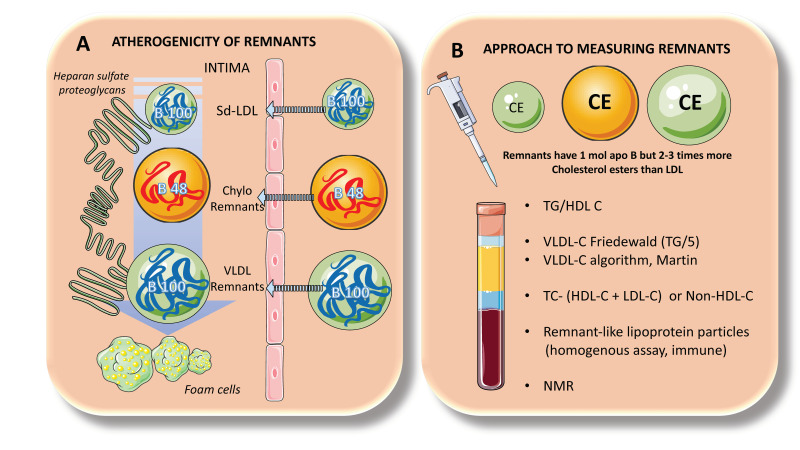
**TRL remnants clinical tips: atherogenicity and analytical approach.** The role of the postprandial phase in atherogenesis cannot be overemphasized. (**A**). In fact, as pointed out earlier, delays in the turnover of TRL remnants produce particles that, as shown to the left, can enter the artery wall and bind to heparan sulfate proteoglycans. Together with LDLs, apo B48 as well as apoB100 TRL remnants can accumulate in the arterial intima and result in the production of foam cells. Indeed, remnants, although less concentrated than LDLs can carry two or three times more cholesterol per particle. The take-home message is that TGs are just a marker for the presence of cholesterol-enriched particles which are per se atherogenic. From a molecular as well as pathological standpoint, TGs are neutral. (**B**). Since TRL remnants are so heterogeneous, so far there is no gold standard for their measurement in clinical practice. However, several practical approaches should be taken into consideration, as shown in the figure to the right. TG/HDL-C remains to be the cheapest way to ascertain the presence of metabolic syndrome dyslipidemia and a surrogate marker for the presence of small-dense LDLs. The Martin algorithm and others are more useful than the classical Friedewald formula to estimate VLDL cholesterol and remnants. Non-HDL cholesterol is also another approximation to the problem and so are new assays for remnant-like lipoprotein particles that are immunological in nature. NMR is not available to everybody and has some pitfalls anyway. The figure was partly generated using Servier Medical Art, provided by Servier, licensed under a Creative Commons Attribution 3.0 unported license.

Lowering LDL-C levels is known to minimize the risk of ASCVD, and it is well accepted that hypercholesterolemia causes atherosclerosis. However, as stated in our introduction, only up to half of ASCVD incidents can be avoided using cholesterol-lowering medication. Recent developments in genetics suggest that increased plasma triglyceride levels may be connected to a significant portion of the “residual risk” of ASCVD [18,19,20]. A consensus view has formed that the higher risk is due, at least in part, to the greater quantity of cholesterol-enriched TRL remnants particles, since triglycerides per se are not known to contribute to atherogenesis, as we previously described. Indeed, smaller chylomicrons and VLDL remnants can and do permeate the artery wall, whereas chylomicrons and large VLDLs cannot, due to their size (Figure 4 and Figure 9A). Even while residual particles contain more TGs than cholesterol, they may nevertheless hold up to twice as much cholesterol per particle as LDLs, due to their larger size. Uncertainty persists over the “quantitative atherogenicity” of remnants in relation to LDLs, but they are considered at least as atherogenic as LDLs [7,31]. In the case of VLDL remnants, these consist of a group of particles known as intermediate-density lipoproteins (IDLs), with a density (d) of 1.006–1.019 g/mL and an analytical ultracentrifuge flotation rate (Sf) of 12–20, which has been demonstrated to overlap in distribution with small VLDL particles with d 1.006 g/mL and Sf 20–60. Although there are only a few unmodified nascent VLDLs and chylomicrons present at any given time due to the lipoprotein lipase quick activity, all particles in this density range (d 1.006 g/mL) contribute to the remnant spectrum to variable degrees [7,31]. 

Remnant particles are hydrolyzed further by hepatic lipase during passage through the hepatic sinusoids and gain additional apoE, which facilitates their binding and uptake by proteins on the surface of the liver cells, such as the LDL receptor (LDLR), LDL-like receptor protein-1 (LRP-1), and the heparan sulfate proteoglycan syndecan-1 (Figure 4 and Figure 6). Additionally, their apoAV content encourages receptor-mediated remnant absorption (Figure 8). Remnant lipoproteins may also bind to and be taken up by the VLDL receptor that is present in extra-hepatic organs [69].

In particular, the bigger size of chylomicron remnants and the lesser surface area of apoB48 vs. apoB100 when compared to VLDLs allows their binding of more apoE and, thus, higher hepatic absorption and shorter plasma residence time under normal metabolic circumstances [72,73,77]. Plasma chylomicron metabolic products typically remain relatively large, lipid-rich, and buoyant, but as VLDL remnants are gradually broken down, IDL particles are created, which then advance to LDLs (Figure 4).

The ability of cholesterol-enriched remnant lipoproteins to transfer their lipid cargo to macrophages—the cells responsible for cholesterol buildup in arterial plaques—is a key characteristic contributing to an elevated risk of ASCVD (Figure 9). This is why it is vital to note that a remnant particle may transport more cholesterol than an LDL particle. Remnant lipoproteins that have undergone oxidative modification play a significant role in atherogenesis due to their pro-inflammatory effects, which are more pronounced per particle than those of modified LDLs, as well as their uncontrolled absorption by macrophage scavenger receptors.

Remnant lipoproteins also carry thrombogenic factors, activate monocytes, and promote endothelial cell death and smooth muscle cell proliferation, among other atherogenic characteristics. Their higher levels of apoCIII may also have atherogenic effects because they have been demonstrated to facilitate monocyte adhesion to endothelial cells and to trigger inflammation through alternate inflammasome activation [1,30,31]. Both apoCIII and apoCI enrichment slow down receptor-mediated hepatic uptake of remnants, and there is evidence that apoCIII inhibition of uptake is caused by mechanisms involving LRP-1 and LDLR. However, as stated before, this is more important in rodents than in humans. Remnants are prone to further modifications because of their plasma residence time being prolonged, which can increase their atherogenicity. Finally, smaller, dense LDL are produced as a result of the breakdown and intravascular remodeling of bigger VLDL particles, such as VLDL1 of Sf 60, which has atherogenic features that intensify the vascular effects of its remnant precursors (Figure 4, Figure 5 and Figure 9).

## 8. Measurement of TRL Remnants

Due to their similar size and density, TRL remnants cannot be distinguished from fewer lipolysis-degraded TRLs using conventional laboratory techniques. However, as most circulating chylomicrons and VLDLs are only partially lipolyzed, overall plasma triglyceride concentration can be appreciated as a general, non-specific proxy for the total concentrations of remnant lipoproteins. The pathogenic significance of increased cholesterol cargo inside remnant lipoproteins, on the other hand, offers the justification for ASCVD risk assessment utilizing tests that measure plasma levels of remnant lipoprotein-cholesterol. Clinical laboratories have offered this measurement in the fasting state using formulae that extrapolate the VLDL cholesterol concentration from the plasma total triglyceride level (Figure 9B).

Plasma triglyceride/5 has historically been the most often used formula for determining VLDL cholesterol, which was developed by Friedewald et al. Its usage, however, is restricted to triglyceride levels below 400 mg/dL, necessitating the direct measurement of cholesterol in the ultracentrifugally separated d 1.006 g/mL fraction for higher triglyceride levels. However, in recent years, algorithms that more correctly reflect variation in VLDL-cholesterol content as a function of plasma triglyceride level have replaced the Friedewald equation. One of the most popular algorithms now being used by clinical laboratories is that of Martin et al. [15,37,69,71,90,91,92,93].

The subtraction of HDL-cholesterol and LDL-cholesterol (properly determined by direct assay) from total cholesterol is a surrogate measure of remnant cholesterol that is especially pertinent to the non-fasting state. However, as the typical LDL cholesterol assay includes the cholesterol in IDL as well as LDL particles, this metric does not account for the cholesterol present in the IDL region of the remnant lipoprotein spectrum [37,69,91].

“Remnant-like” lipoprotein particles (RLP), which are a subcategory of remnants, can be measured by immunoseparation-based methods. However, the nature of the apoB100 epitope used in this assay has raised questions, and it has been demonstrated that the cholesterol-rich component of RLP includes a significant contribution from LDL particles, especially in people with lower plasma triglyceride levels [7].

Nuclear magnetic resonance spectroscopy is an alternate method for measuring cholesterol in particles in the remnant lipoprotein size range, but questions about the reliability of such an analytical system have been raised.

In a nutshell, there is currently no ideal method for measuring remnant lipoprotein plasma concentrations, in large part because there is no acknowledged standard for characterizing remnant lipoproteins or their atherogenic components. As we wait for further developments in the analysis of these complex particles, a practical clinical approximation to TRL remnants can be assessed by using some of the formulae and methods shown in Figure 9B.

## 9. TG-Lowering Therapy

In agreement with what was expressed in this review so far, we should first qualify what we mean when we speak of triglyceride-lowering therapy. The objective of the therapy is not to lower TGs per se but to lower the amount of circulating TRL remnants. With that in mind, we can therefore continue with this discussion. The first step in producing positive changes in plasma triglyceride levels is adopting a healthy diet. First, it is important to consume less sugar and processed carbohydrates while increasing the intake of wholegrain foods and dietary fiber. Secondly, foods high in monounsaturated and polyunsaturated fats should provide most of the calories from fats. Thirdly, controlling total calorie consumption is crucial for achieving and maintaining a healthy weight.

Further research is needed to understand how triglyceride transport is regulated and the metabolic abnormalities that cause remnant accumulation and increase the risk of ASCVD. The novel therapeutics that use antibody and RNA-silencing technologies are highly specific and exhibit improved triglyceride-lowering properties, in contrast to the previous generation of triglyceride-lowering medications (including fibrates, nicotinic acid, and omega-3 PUFAs), which had a broad range of effects but limited efficacy.

**Figure 10:**
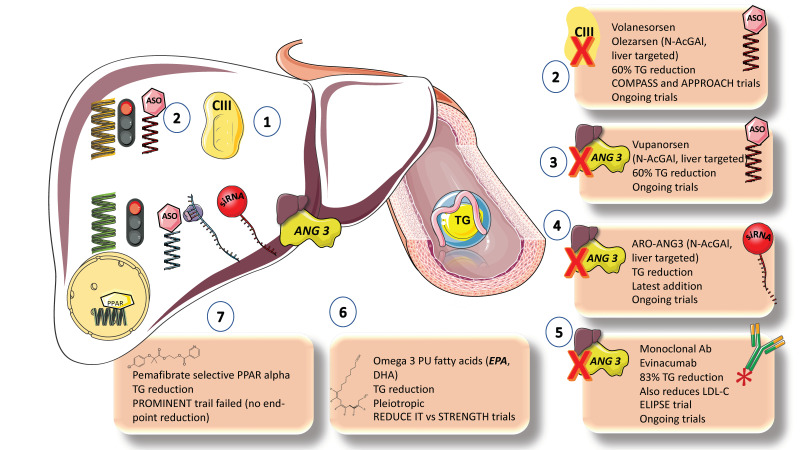
**Main therapeutic options (current and in the pipeline) for TRL dyslipidemia beyond lifestyle changes.** In the past few years, elegant research on animals and loss of function studies in humans has earmarked both apoCIII and ANGPTL3 as pharmacological targets. Monoclonal antibodies and inhibitors of their translation have been developed and have gone through phase one to three trials, in the beginning especially for familial hypertriglyceridemias. (1) For ApoCIII, an antisense oligonucleotide (ASO), volanesorsen (2) proved very effective to reduce TGs but had side effects. The new liver-targeted Olezarsen has fewer side effects, and it is very effective. Several trials are ongoing. ANGPTL3: Three approaches have been developed for the inhibition of ANGPTL3, ASO, small-interference RNA (siRNA), and monoclonal antibody (mab). (3) Vupanorsen is an N-AcGal liver-targeted ASO. (4) ARO-ANG3 is an N-AcGal liver-targeted siRNA, the latest addition to the armamentarium and (5) Evinacumab is a promising monoclonal antibody that provides up to 83% reduction in TGs and reduces LDL cholesterol. Ongoing trials for the three approaches are taking place. Other approaches beyond lifestyle changes are the classics (6) Omega-3 fatty acids, whose mode of action is pleiotropic. EPA has proven to be effective in residual risk reduction in cardiovascular disease through the REDUCEIT trial with some controversy with the results from the STRENGTHS trial. (7) PPAR alpha inhibitors. The selective PPAR alpha inhibitor Pemafibrate has just failed to provide an end-point reduction in cardiovascular disease residual risk management, as shown by the PROMINENT trial. The figure was partly generated using Servier Medical Art, provided by Servier, licensed under a Creative Commons Attribution 3.0 unported license.

Statins, fibrates, ezetimibe, niacin, PCSK9 inhibitors, and omega-3 fatty acid preparations are among the medications that can lower plasma triglyceride and/or LDL-C levels (Figure 10).

- Statins, PCSK9 inhibitors, and ezetimibe have only modest effects on triglyceride levels (usually a 5–15% reduction), whereas niacin, fibrates, and omega-3 fatty acids are considered moderately effective, causing triglyceride levels to drop by up to 40% in patients with severe hypertriglyceridemia [2,5,20,94,95].
- Fibrates. Although the FIELD and ACCORD trials in patients with type 2 diabetes did not show a significant beneficial effect of fenofibrate on ASCVD outcomes, subgroup analyses showed that patients with elevated triglyceride concentrations and/or low HDL-C levels have a significantly lower risk of ASCVD. Pemafibrate, a selective PPAR agonist, is not protective against ASCVD in patients with elevated triglyceride levels and type 2 diabetes, according to the recently completed large PROMINENT trial. This finding should put to rest a long-standing debate about the clinical advantages of this therapeutic approach [96].
- VLDL production inhibitors. The metabolic processes outlined above can be used to develop two basic strategies for reducing TRLs and remnants: decreasing hepatic production of VLDL1 and increasing clearance of TRLs by boosting lipolysis and encouraging remnant uptake by the liver. By inhibiting apoB-100 synthesis or limiting the availability of TGs for VLDL particle assembly, VLDL1 production can be reduced. Omega-3 PUFAs, the MTTP inhibitor lomitapide, and mipomersen (an antisense oligonucleotide inhibitor of apoB synthesis) are medications that are readily available and have been shown to reduce the generation of VLDL1. However, because lomitapide and mipomersen have a well-documented negative effect of promoting lipid accumulation in the liver, which can lead to NAFLD, their effectiveness has not been evaluated in outcomes trials [85].
- Omega 3 FAs. With the notable exception of the REDUCE-IT trial of high-dose eicosapentaenoic acid (EPA), which reported a 28% relative risk reduction, omega-3 PUFA preparations have been tested in large-scale ASCVD outcome studies that, overall, have only provided weak evidence of their benefits in reducing ASCVD risk. The STRENGTH study did not replicate this benefit. These conflicting results have given rise to an ongoing debate about the fundamental mechanism underpinning the benefits of EPA shown in REDUCEIT. Numerous studies published over the past five years raise the possibility that the use of mineral oil as the placebo comparator in the REDUCEIT trial may have contributed in a small way to the perceived risk reduction in the EPA group compared to the control group [10].
- New targets, blocking inhibitors of LPLs [10,28,33,97,98,99]. In lipid-lowering medications over the past five years, attention has been placed on developing substances that boost TRL lipolysis. ApoCIII and ANGPTL3 are now the main targets due to their understood roles in regulating LPL activity and the availability of genetic information that supports the proof of concept that lowering the concentration of these targets is likely to result in reduced ASCVD risk. Monoclonal antibody inhibition and RNA silencing technologies are the two most promising approaches. Antisense oligonucleotides targeting APOC3 mRNA (volanesoren and olezarsen) and ANGPTL3 mRNA (vupanorsen) are now being tested in phase II and phase III clinical trials [100,101]. These compounds reduce plasma levels of apoC-III by 70–80% and ANGPTL3 by roughly 80% in both animal and human studies. In patients with homozygous familial hypercholesterolemia, evinacumab, a monoclonal antibody targeted against ANGPTL3, has been shown to reduce plasma triglyceride and LDL-C levels. This finding suggests a different mode of action for LDLs than other medications that increase LDL receptor activation [28,33,77,97,98,99].

## 10. Conclusions

Triglycerides (TGs) are surrogate markers of the catabolic residues of TRL (remnants) which often bear twice as much cholesterol as LDLs. Triglyceride catabolism is mostly to blame for the buildup of circulating TRLs and their remnants. It is believed that these residual particles, which are high in cholesterol, cause atherogenesis. We presented an overview of the key pathways of TRL metabolism, emphasizing the recently discovered role of apolipoproteins, the critical physiological role of lipoprotein lipase and its primary regulators, and the significance of these particles’ fluxes in the postprandial period. In order to better understand how TRLs are distributed during the fast-fed cycle, we looked at their catabolic rates and the key roles of apoCIII, apo AV, and angiopoietin-like protein functions. With a final outlook on the various methodological ways to assess TRL remnants, still in quest of the gold standard, we present a brief synopsis of the new and old treatment arsenal, the results of significant current trials, and other pertinent information.

Among the many questions that remain to be answered and will surely spark intensive research in the following years, the following have been posited [25,26]:

1. What governs the ultimate integration between the apoCIII, apoAV, and ANGPTL regulatory actions on LPL activity?
2. What additional hormonal and dietary factors affect the activity of ANGPTL3, ANGPTL4, and ANGPTL8, and what long-term effects might be expected if their regulation is pharmacologically disrupted?
3. How should patients with hypertriglyceridemia control postprandial lipemia?
4. Are there other factors that control the production of remnants?
5. How important are receptor abundance and apolipoprotein composition in influencing the rate of remnant clearance by the liver?
6. Role of microbiota;
7. Role of brain-gut axis and incretins in chylomicron metabolism;
8. Analytically speaking, is there a proteomic or lipidomic ‘signature’ that characterizes TRL remnant particles that would make precise quantification possible?

## Data Availability

Not applicable.
